# Supplementary material for: Mathematical models of drug-resistant tuberculosis lack bacterial heterogeneity: A systematic review
Source: PLoS Pathog. 2024 Apr 10;20(4):e1011574. doi: 10.1371/journal.ppat.1011574 (PMC11060536; doi:10.1371/journal.ppat.1011574)
Supplement: S1 Text — (DOCX) [file ppat.1011574.s001.docx]

Mathematical models of drug-resistant tuberculosis lack bacterial heterogeneity: a systematic review

Naomi M. Fuller, Christopher F. McQuaid, Martin Harker, Chathika K. Weerasuriya, Timothy D. McHugh, Gwenan M. Knight

**S1 Text**

The search in all five databases was conducted following the same strategy; an example for the Medline database is shown below. Where subject headings were available, they were used (Medline, Embase and Global Health). The library at LSHTM checked all searches to ensure the correct methodology was being used.

**Ovid (Medline ALL)**

keyword search + subject headings search

1 ((comput* or math* or compart* or stochastic or statist* or deterministic or transmission or dynamic* or population or theor*) adj5 model*).mp.

2 exp Models, Theoretical/ or Computer Simulation/

3 1 or 2

4 (drug-resistan* or DR or multidrug-resistan* or multi-drug resistan* or MDR or extensive drug-resistan* or XDR or antimicrobial resistan* or anti-microbial resistan* or AMR or antibacterial resistan* or anti-bacterial resistan* or ABR or antibiotic resistan* or heteroresistance).mp.

5 drug resistance/ or drug resistance, microbial/ or drug resistance, bacterial/ or drug resistance, multiple, bacterial/ or drug tolerance/

6 4 or 5

7 (Tuberculosis or TB or mycobacteri* or antimycobacterial or antitubercular).mp.

8 Mycobacterium/ or mycobacterium infections/ or mycobacterium infections, nontuberculous/ or tuberculosis/ or tuberculosis, multidrug-resistant/ or extensively drug-resistant tuberculosis/ or exp antitubercular agents/ or antibiotics, antitubercular/

9 7 or 8

10 3 and 6 and 9
