# Supplementary material for: Mathematical models of drug-resistant tuberculosis lack bacterial heterogeneity: A systematic review
Source: PLoS Pathog. 2024 Apr 10;20(4):e1011574. doi: 10.1371/journal.ppat.1011574 (PMC11060536; doi:10.1371/journal.ppat.1011574)
Supplement: S2 Text — (DOCX) [file ppat.1011574.s002.docx]

Mathematical models of drug-resistant tuberculosis lack bacterial heterogeneity: a systematic review

Naomi M. Fuller, Christopher F. McQuaid, Martin Harker, Chathika K. Weerasuriya, Timothy D. McHugh, Gwenan M. Knight

**S2 Text**

Details of extraction table for Stage 1

We extracted the following information from each of the papers deemed to capture DR-mycobacteria. A working rule was only to extract from papers where it was possible to extract the following information.

- **Paper information**: Publication date, Authors and Title
- **Status:** Whether it should be included in the full-text extraction (Stage 2)
- **Simulation/Mathematical model**: Whether the paper contained a model
- **Model characteristics:**

Type: we chose to use six categories here. Models of human populations with transmission of bacteria were defined as either a (1) “transmission” model which dynamically accounts for the spread of bacteria between individuals or populations (number of new infections is dependent on how many are currently infected) or (2) “state transition” models where individuals or populations move between different disease states and the force of infection is a static input parameter (number of new infections is NOT dependent on how many currently infected). (3) “Decision analytic” models captured a cohort of individuals, typically moving through a treatment or diagnostic pathway with usually no ongoing transmission or only including fixed transmission rates in sensitivity analysis. These were differentiated from (4) “bacterial dynamics” models that captured bacterial populations only. The other model type included was (5) pharmacokinetic / pharmacodynamic (“PK/PD”) which captures the concentrations and effect of drugs *in vivo* that include parameters related to bacterial populations. We also had models that were combinations of two types of modelling techniques such as “transmission + state transition”, “transmission + operational” or “state transition + statistical”. Here “operational” is defined as a discrete-event simulation for patient pathways diagnostic procedures, and “statistical” is defined as a model used to predict information in an idealised format based on fitting to population data.

Aim: Our models fell under seven categories. Those that modelled (1) “interventions” without any consideration of economic impact and (2) those that explored costs (“interventions + cost-effectiveness”). Due to the focus on drug resistance, we separated out interventions that explored any chemotherapy (e.g. different drug dosing or treatment regimens) for mycobacteria (“treatment interventions”) (3) and of these, models that looked at linked costs to drug changes (“treatment interventions + cost-effectiveness”) (4). These two categories of “treatment interventions” included PK/PD models and those of new treatment regimens, as well as antibiotic preventative therapy (such as IPT) but not necessarily of HIV treatment interventions.

We then separated out those that estimated “burden estimation” – these models aimed to provide a quantification of how many individuals were / are / could be infected with resistant *M.tb* or of the route to infection (e.g. how many DR-*M.tb* transmitted vs. *do novo* generation) or explored burden estimation (5).

Models that theoretically explore the interactions between susceptible and resistant strains to, for example, determine stability thresholds are labelled as “theoretical” (6). This also includes models that explore intervention impact on theoretical results such as stability.

Those that aimed to estimate parameters (e.g. fitness costs to resistance or acquisition rates), potentially by comparing to data, trends or the impact of varying model structures or components are labelled “parameter estimation” (7).

Setting: For models of human populations, this could be at the global, WHO geographical region, country, or specific subset of the population level (such as prison population). Here, the global setting was used if results of individual country or region-level models were combined to give a final result at the global level. For models of bacterial populations, this could be either modelling of *in vitro* laboratory data or within-host dynamics such as PK/PD models linked to immune responses.

Species: We aimed to capture what specific bacteria in the Mycobacterium genus were being modelled (1) and if there were any additional pathogens (2) or diseases (3) being modelled such as HIV or diabetes.

Resistance: We aimed to capture data on all resistances that have been modelled and so recorded two types

(1) Hypothetical: For models not specific to a disease such as TB; if mono- or multiple-resistances have been captured in the model of hypothetical mycobacteria populations without specifying the antibiotics modelled

(2) Specific: For models specific to a disease such as TB; if the model captures resistance to specific drugs with associated parameters / data. Common resistances of mono-resistance to isoniazid or rifampicin and resistance to isoniazid and rifampicin (termed as MDR-) were recorded as well as details of more resistance levels. If a paper modelled rifampicin-resistance as a proxy for or interchangeably with MDR, we included this in the MDR/RR- category, i.e. a patient with a strain found to be rifampicin resistant would be treated as an MDR TB patient and trigger use of second-line therapies.

We did not distinguish between models that looked at one resistance and then another resistance separately (e.g. looked at the spread of isoniazid mono-resistance and then separately as rifampicin mono-resistance – this would be coded as a paper that looked at isoniazid and rifampicin mono-resistance in the same way as a paper that modelled both at the same time).

- **Reasons for removal**
  - Removal from stage 1 extraction: Papers that were not deemed to capture DR-mycobacteria were removed from stage 1 of the extraction with the following categorisations for removal:
- Is the publication in English?: If no, remove and classify as “not English”, if yes, proceed to next question.
- Is the publication a journal article?: If no, remove and classify as “not a paper”, if yes, proceed to next question.
- Does the paper contain a mathematical model (as defined in the methods section)?: If no, remove and classify as “no model”, if yes, proceed to next question.
- Does the model include a population of Mycobacterium?: If no, remove and classify as “no MB”, if yes, proceed to next question.
- Does the model include Mycobacterium with antibiotic resistance?: If no, remove and classify as “no resistance”, if yes, proceed to next question.
- If there is a host, is it human only?: If no, remove and classify as “non-human host”, if yes, proceed with stage 1 extraction.
  - Removal from stage 2 extraction: Papers that were not deemed to capture DR-mycobacteria with bacterial heterogeneity were removed from stage 2 of the extraction with the following categorisation for removal:
- Does the model capture bacterial heterogeneity (as defined in the methods section)?: If no, remove and classify as “no bacterial heterogeneity”, if yes, process with stage 2 extraction.
