# Supplementary material for: Mathematical models of drug-resistant tuberculosis lack bacterial heterogeneity: A systematic review
Source: PLoS Pathog. 2024 Apr 10;20(4):e1011574. doi: 10.1371/journal.ppat.1011574 (PMC11060536; doi:10.1371/journal.ppat.1011574)
Supplement: S3 Text — (DOCX) [file ppat.1011574.s003.docx]

Mathematical models of drug-resistant tuberculosis lack bacterial heterogeneity: a systematic review

Naomi M. Fuller, Christopher F. McQuaid, Martin Harker, Chathika K. Weerasuriya, Timothy D. McHugh, Gwenan M. Knight

**S3 Text**

Details of extraction table for Stage 2

From each of the papers deemed to capture DR-mycobacteria with bacterial heterogeneity, we extracted the following information:

**Paper information:** Publication date, Authors, and Title

**Model information:** Model_type: as for Stage 1

Deterministic or stochastic: If the model included any random processes, then we termed it stochastic. If analysis explored deterministic models, followed by more complex stochastic models we said that the paper used “both”.

ODE: If the model contained ordinary differential equations, (1) “1” for yes and (2) “0” for no.

Methodology: the aim of this section was to record what mathematical methodologies were used to construct the model so that methodologies could be repeated if wished, free text, subjective

**Data information (we aimed to capture data used in models but also methods of using said data):**

Data origin: Data used to validate models could either be obtained from (1) “primary data” where the authors had generated their own data or (2) “Literature” data where the data was sourced from the literature.

Data type: Data used in the models may fall into different categories with some models using a combination of categories, (1) “Clinical” data was sourced from clinical trials or studies, (2) “Epidemiology” data came from any epidemiology studies such as case-control studies, cohort studies or mathematical modelling of an epidemiologic question and extends to include population level information such as demographics, (3) “WHO” data differs from the previous category as it refers to any data sourced directly from the WHO, which is often epidemiology data, however as WHO data is often a statistical combination or prediction based on a range of input data and it is used frequently in TB models it is treated as a separate data type, (4) “experimental” data was from experiments conducted with *Mycobacteria* in a laboratory setting (5)”

**Setting information (we aimed to capture where models were set and the context they were situated in):**

Public health question: We distilled the aim of the paper into one question, so it was easily summarised for us and to spot any trends in public health questions asked.

Aim: same as for stage 1

**Population information (we aimed to capture what population type the model was simulating):**

Population type: We expected there to be two population types modelled in mycobacteria models (1) “human” population models where human hosts infected with a mycobacterial population were the main focus and (2) “bacterial” population models where resistant and non-resistant bacteria were the main focus

Population setting: We found that the target population could be set in different circumstances such as (1) “Within host” where a bacterial population is being modelled within a human host, (2) “Laboratory” where a bacterial population is being modelled with a laboratory setting, or (3) “community” where infected human hosts are modelled in a non-specific setting, (4) “hospital” where infected human hosts are modelled in a hospital setting or (5) free text for another setting which could be a household or prison or other setting

Immune system: We wanted to record if any models included the immune system such as compartments for immune response, host killing rates or macrophages for example, (1) “1” for yes and (2) “0” for no.

MB: Record what mycobacterial species the model is simulating

Other: Record other diseases such as HIV or diabetes that are also included in the model.

**Resistance information (how was resistance captured in the model)**

Acquired or transmitted: resistant TB can be either (1) “acquired” where resistant mycobacterial strains arise in a susceptible mycobacterial population or (2) “transmitted” where resistant mycobacterial strains pass from host to host (human models only)

Discrete or continuous: antibiotic resistance exists as a continuum in a bacterial population, so we wanted to capture if models included resistance as this continuum (1) or if they used multiple discrete (2) definitions that match clinical categorisations.

Transmission affected: We wanted to record if resistance in the model affected the transmission of resistant bacteria compared to susceptible bacteria, (1) “1” for yes and (2) “0” for no.

Transmission how: Free text to record the mathematical application of transmission difference.

Disease progression affected: We wanted to record if resistance in the model affected the disease progression of resistant bacteria compared to susceptible bacteria, (1) “1” for yes and (2) “0” for no. Examples of this could include mutational rates, resistant specific latent states and fitness for bacterial growth models only.

DP how: free text to record the mathematical application of disease progression difference.

Operational affected: We wanted to record if resistance in the model affected the operational effects of resistant bacteria compared to susceptible bacteria (1) “1” for yes and (2) “0” for no. Examples of this included treatment success and disease detection.

Ops how: free text to record the mathematical application of operational difference.

**Heterogeneity information:**

Fitness: did the model include a different fitness for antibiotic resistant bacteria from antibiotic susceptible bacteria? (1) “1” for yes and (2) “0” for no.

Fitness how: we recorded how fitness is calculated and used in the model, free text

Mutation rate: did the model include a rate at which mutations that confer resistance occur in a bacterial population? (1) “1” for yes and (2) “0” for no.

Resistance acquisition: we recorded how resistance was developed or acquired in the model as not all models had a base mutation rate

Population stratification: we recorded what characteristics of a population were used to separate the population into different categories with different characteristics. These differences could be reflected by parameters changes for as resistance, fitness, metabolic state or natural history

Natural history: we recorded how, if any, natural history states of the disease were included in the model, such as latency. Free text

Pop_strat_details: free text to record specifics of how population stratification was implemented in the model, free text

**Output of interest**: We wanted to know what was the main mathematical output that the model was aiming for, e.g. difference in growth rate

**Conclusions:** We wanted to know how heterogeneity affected the outcome and conclusions of the model (free text, subjective)
