# Supplementary figures and images for: Mathematical models of drug-resistant tuberculosis lack bacterial heterogeneity: A systematic review
Source: PLoS Pathog. 2024 Apr 10;20(4):e1011574. doi: 10.1371/journal.ppat.1011574 (PMC11060536; doi:10.1371/journal.ppat.1011574)

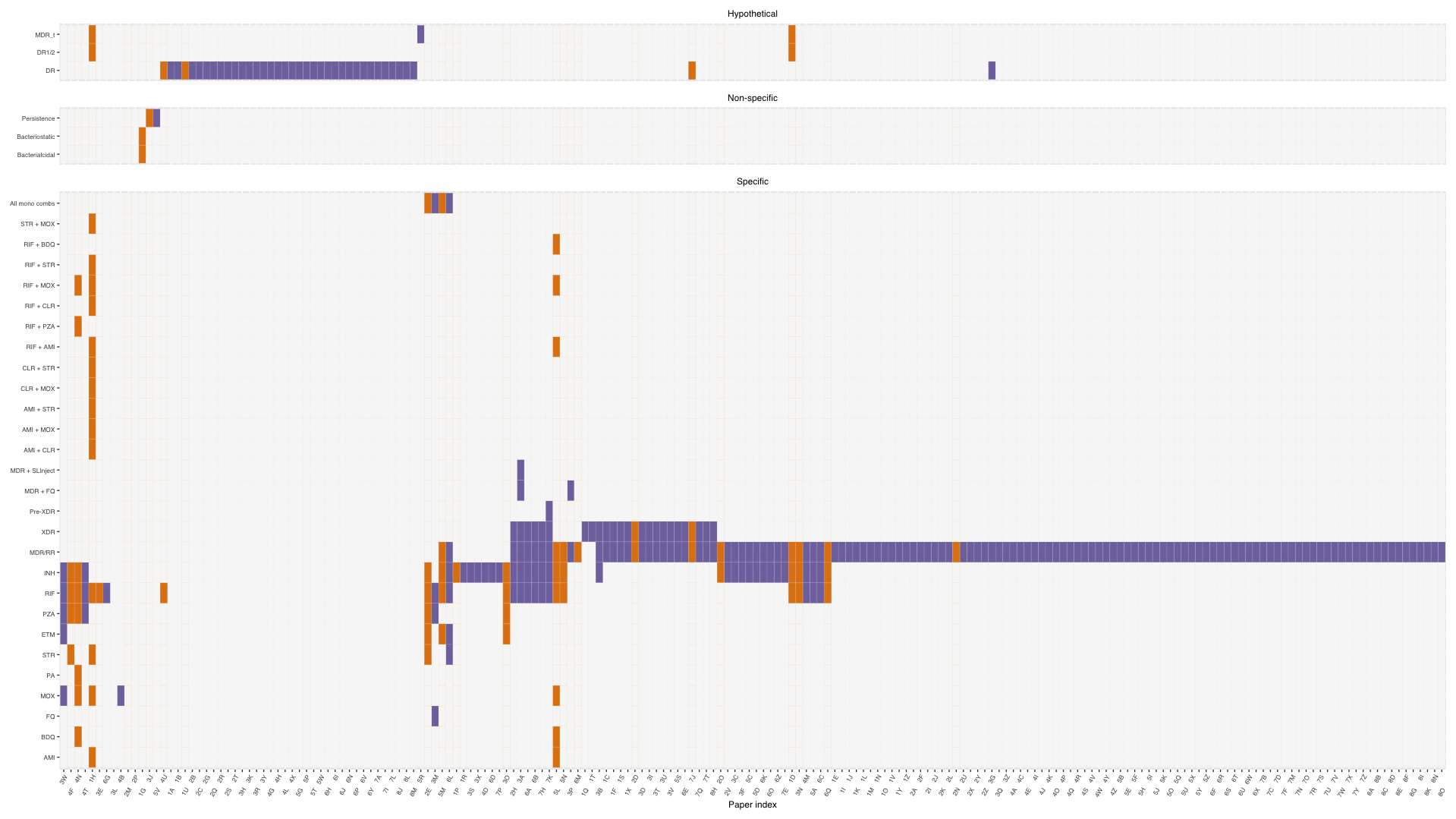

Supplement: S1 Fig — Heatmap of resistances included per DR-TB model (n = 195) indicates a lack of diversity in resistances modelled, with MDR/RR-TB featuring in over half of all 195 models. Each coloured line indicates a model (y axis) included in stage 1 (purple) or stage 2 (orange). The graph groups models into specific (captures resistance to a named antibiotic), non-specific (defined resistance that are not specific to an antibiotic) and hypothetical (captures antibiotic resistance not linked to a named drug). Antibiotic acronyms as follows: AMI = amikacin, BDQ = bedaquiline, CLR = clarithromycin, ETM = ethambutol, FQ = undefined fluroquinolone, LZD = linezolid, MOX = moxifloxacin, PA = pretomanid, PZA = pyrazinamide, STR = streptomycin, INH = isoniazid, RIF = rifampicin, MDR/RR = multidrug resistant/ rifampicin resistant, XDR = extensively drug-resistant, SLInject = second line injectable antibiotic (from WHO guidelines 2014), another 1st line = rifampicin, ethambutol, or pyrazinamide. Index links to paper number in S1 Table. (DOCX) [file ppat.1011574.s004.docx]

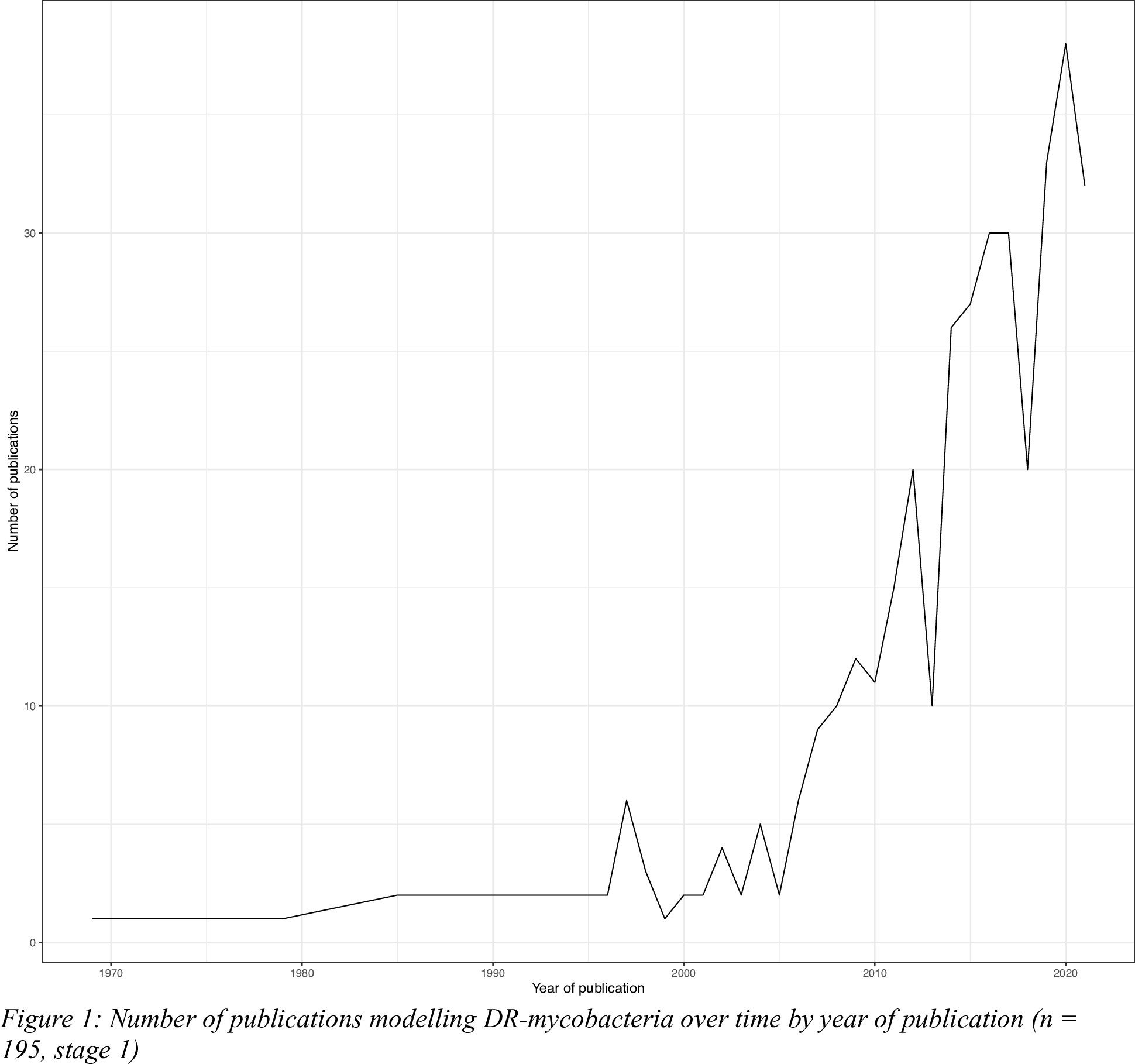

Supplement: S2 Fig — (TIF) [file ppat.1011574.s005.tif]
