# Supplementary material for: Mathematical models of drug-resistant tuberculosis lack bacterial heterogeneity: A systematic review
Source: PLoS Pathog. 2024 Apr 10;20(4):e1011574. doi: 10.1371/journal.ppat.1011574 (PMC11060536; doi:10.1371/journal.ppat.1011574)
Supplement: S3 Table — (DOCX) [file ppat.1011574.s008.docx]

**S3 Table**

119 models were identified in stage 1 that had a geographical setting. The number of models found in each geographical setting is shown here.

| Settings | Number of models |
| --- | --- |
| Angola | 1 |
| Asia-Pacific | 1 |
| Australia | 1 |
| Azerbaijin | 1 |
| Bangladesh | 7 |
| Belarus | 3 |
| Bhutan | 1 |
| Botswana | 4 |
| Brazil | 1 |
| Brunei | 1 |
| Bulgaria | 1 |
| Cambodia | 1 |
| China | 15 |
| community + institutional amplifier | 1 |
| Cuba | 1 |
| Democratic Republic of the Congo | 2 |
| Dominican Republic | 1 |
| Eastern Europe | 1 |
| Ecuador | 1 |
| Egypt | 1 |
| Estonia | 3 |
| Ethiopia | 4 |
| Gambia | 1 |
| Germany | 3 |
| Global | 7 |
| High HIV Setting | 1 |
| High MDR Setting | 1 |
| High-Incidence Setting | 2 |
| Hospital | 1 |
| India | 25 |
| Indonesia | 7 |
| Kazakhstan | 1 |
| Kenya | 3 |
| Kyrgyzstan | 1 |
| Laos | 1 |
| Lesotho | 2 |
| Low-Incidence Setting | 2 |
| Malawi | 2 |
| Malaysia | 1 |
| Maldives | 1 |
| Med-Incidence Setting | 1 |
| Mines | 1 |
| Moldova | 2 |
| Mozambique | 1 |
| Myanmar | 3 |
| Namibia | 1 |
| Nepal | 1 |
| Nicaragua | 1 |
| Nigeria | 2 |
| North Korea | 3 |
| Pakistan | 1 |
| Papua New Guinea | 3 |
| Peru | 6 |
| Philippines | 7 |
| Prisons | 4 |
| Roma | 1 |
| Russia | 8 |
| Singapore | 1 |
| Somalia | 1 |
| South Africa | 22 |
| South East Asia | 4 |
| South Korea | 1 |
| Sri Lanka | 1 |
| Swaziland | 5 |
| Taiwan | 2 |
| Tajikistan | 1 |
| Tanzania | 2 |
| Thailand | 5 |
| The Netherlands | 1 |
| Timor-Leste | 2 |
| UK | 3 |
| Ukraine | 1 |
| USA | 4 |
| Uzbekistan | 3 |
| Venezuela | 1 |
| Vietnam | 6 |
| Zimbabwe | 3 |
