# Supplementary material for: Mathematical models of drug-resistant tuberculosis lack bacterial heterogeneity: A systematic review
Source: PLoS Pathog. 2024 Apr 10;20(4):e1011574. doi: 10.1371/journal.ppat.1011574 (PMC11060536; doi:10.1371/journal.ppat.1011574)
Supplement: Attachment — Submitted filename: Responses to Reviewer Questions.docx [file ppat.1011574.s009.docx]

Reviewer's Responses to Questions

**Part I - Summary**
Please use this section to discuss strengths/weaknesses of study, novelty/significance, general execution and scholarship.

Reviewer #1: This paper provides a systematic overview of TB models which address questions related to drug resistance. The authors include a 2-stage review where they first identify within-host models and transmission models that consider questions related to drug resistance, then identify the subset of these models that allow for variation of resistant isolates (for the within-host models) or patients with resistant TB (for the transmission models).

*Thank you for taking the time to review this manuscript as thoroughly as you have. We appreciate the comments you have made and have addressed them. We feel the manuscript is considerably stronger now, thanks to your feedback. We have responded to each comment in italics below and referenced the line number in the manuscript version with track changes where necessary. We have made alterations to Figures 3, 4 and 5 in line with the comments provided here. We also altered Figure 1 so that the boxes with the excluded number of papers read the same, and Figure 2 has been altered for a higher pixel density.*

The manuscript is useful in that provides a timely summary of the literature on modeling of DR-TB, with a specific question about how bacterial heterogeneity is handled (or omitted) from these models. A major challenge involved in this review is for the authors to provide clear description of the rationale for inclusion/exclusion of studies, the classification of these studies, and what is actually meant by “fitness” as this is a composite characteristic that may be modeled with a variety of mechanisms. I have some specific comments related to opportunities to improve these aspects of the paper below.

*We have addressed these, especially for the inclusion/exclusion regarding bacterial heterogeneity, by rewriting and adding extra clarity in the introduction (lines 142-168) and methods (lines 214-237). Regarding our use of fitness, we acknowledge your point and have noted that fitness can be defined and used differently depending on the process, assumptions, and population modelled (lines 522-525).*

Further, I think the authors may want to soften the title and some of the language a bit. I agree with the authors that there may be important consequences for omitting consideration of such bacterial variation, but I do not think that every model-based analysis of drug resistant TB necessarily needs to include this type of variation to be useful. I suspect the authors agree with this, but the title sounds unnecessarily judgemental.

*We have taken this on board and altered the title to reflect this. We have also gone through the text and softened the language where it was perhaps too harsh, without changing our meanings (e.g. lines 577-578 now saying “This is despite several models showing how heterogeneity in transmission fitness can affect DR-TB prevalence estimates (8,62–64)”. Instead of “This is despite several models now having shown how ignoring heterogeneity in transmission fitness can affect DR-TB prevalence estimates (8,62–64)” )*

Reviewer #2: This manuscript aims to summarize the approaches used by published mathematical models of human mycobacterial infections (with a focus on tuberculosis) to evaluate whether and how they account for bacterial heterogeneity. This is an impressively comprehensive review of modeling studies, spanning the range from models of bacterial populations to decision-analytic models to evaluate the cost-effectiveness of tuberculosis treatment interventions. That alone makes this work highly valuable for tuberculosis researchers, and modelers in particular. There are a few key aspects of the work that could be improved to make it suitable for publication.

*Thank you for taking the time to review our manuscript, your comments were very helpful in improving it. We have taken them on board throughout the manuscript.*

**Part II – Major Issues: Key Experiments Required for Acceptance**
Please use this section to detail the key new experiments or modifications of existing experiments that should be absolutely required to validate study conclusions.

Generally, there should be no more than 3 such required experiments or major modifications for a "Major Revision" recommendation. If more than 3 experiments are necessary to validate the study conclusions, then you are encouraged to recommend "Reject".

Reviewer #1: Major comments:
1) Lines 131-135 and Lines 171-175 - I am not quite clear on the distinction being made between the types of heterogeneity considered for the i) within-host models vs. ii) transmission models. I assume the goal is to include models of each type when there is structure that allows the same pressure (ie drug treatment) to exhibit variable selection on mycobacteria of the same resistance phenotype (for within-host models) or individuals infected by mycobacteria with the same resistance phenotype (for transmission models)? In my view, being upfront about the ingredients needed to model selection of more fit DR variants provides a clear rationale for why some studies are included and others are not.

*Thank you for highlighting the detail we need to include in our heterogeneity definitions. We have reworded the paragraphs in the introduction and the methods regarding bacterial heterogeneity to address this comment and the comment below. Specifically, in the introduction, we have included the following paragraph.*

“*We envisaged two broad settings of papers to be included in this review, within-host and between-host transmission models. This was noted by Cohen et al. (2009), a previous review of the DR-TB modelling literature (4), where “between-host” models refer to models on the human population scale. Since 2009, there has been an increase in models of bacterial populations set in the laboratory. As the populations captured will be similar to within-host models, we combined laboratory models and within-host models and collectively called them “bacterial population” models.*

*The aims, dynamics and model structure of between-host models differ considerably from bacterial population models, namely by transmission of the pathogen and populations included, making them difficult to compare. Therefore, we defined heterogeneity differently for bacterial populations and between-host models to compare methods within these categories and gain a clearer picture of bacterial heterogeneity modelling. At the between-host level, we were interested in capturing those models that went beyond capturing resistance phenotypes but included any added dimension of bacterial variation, including what may affect survival, such as fitness effects. Models of bacterial populations that captured any resistance variation were included; distinct populations of resistant bacteria needed to be modelled, which differed in their parameter values (e.g. growth rate or mutation rate)*.” (lines 146-169)

*The methods now state: “We split models into two groupings: “between-host” and “bacterial population” models, with the differences in their model scale, structure, and aims, resulting in a different bacterial heterogeneity definition. A “between-host” model was classed as a heterogenous model when strains infecting a human population resistant to the same drug varied in another characteristic such as fitness, rates of compensatory mutation evolution or associated treatment recovery rates. These characteristics were extracted during the full-text extraction stage. “Bacterial population” models included both within-host and models of bacterial populations capturing dynamics measured in laboratory or experimental conditions. A bacterial population model was classed as a heterogeneous model when there were distinct resistant strains captured which had different parameter values such as fitness, mutation rates and metabolic states. These parameter differences were extracted during the full-text extraction stage. ” (lines 214-237)*

2) Relatedly, some of the wording is ambiguous in these sections and should be clarified, for example:
a. Line 133: “strains resistance (sic) to a single drug should also vary in another characteristic.” I assume this should be replaced by individuals infected with strains resistant to the same drug should also vary in another characteristic – that is, you are not interested in models that include INH and Rif monoresist TB of different transmission fitness, but rather models that include Rif resistant TB of variable transmission fitness.
b. Line 134: “multiple resistant strains were categorized and parameterized differently from each other.” I assume this means that bacterial isolates of the same/similar resistance phenotype differed in way (e.g. growth rate)

*Thank you for these very helpful suggestions. We have included them in our updated definitions shown above.*

3) For stage 1 review, I am wondering if the authors collected information about the time horizon modeled in each of the included studies (especially for the transmission models). Given the relatively slows dynamics of TB and DR-TB, I think readers would be interested in understanding the scope of the time horizons that have been modeled.

*That is an interesting dimension that we did not explore. However, we think this will benefit future work and have added the following sentence in the discussion: line 618 “... nor time horizons for each model”*

4) Figure 3: The categories of model types and aims for Figure 3 are confusing. Some of the type categories refer to the scale of the model (e.g. bacterial population) while some refer to the modelling approach (e.g. decision analytics), and some are subcategories of each other. The categories of model aims also seem a bit ad hoc and unclear and potentially overlapping (e.g. burden, data analysis vs theoretical, intervention). I find this difficult to make sense of and wonder if there is a more coherent way to categorize model types and usages for a reader such that the categories are mutually exclusive groups?

*We agree that it is challenging to categorise models and papers so discreetly and that some detail will be lost, and there will be overlap between models. We have added a line in the methods to address this: lines 254-258 “DR-mycobacteria models can address multiple aims with various methods but they will have a common theme such as parameter estimation or impact of interventions. We extracted information from the models to categorise and classify them within five categories, focusing on the main theme of the model.“*

*To clarify Figure 3, we have made some changes to prevent an overlap:*

- *Have changed “bacterial growth” to “bacterial dynamics”*
- *Changed “data analysis” to “parameter estimation”*
- *Changed “burden” to “burden estimation”*
- *Changed “interventions” to “non-treatment interventions”*
- *Added a bracket indicating that “non-treatment interventions” and “interventions”, with and without cost-effectiveness, represent all interventions in the models*
- *Added brackets for model types indicating that the state transition and transmission models are related.*

*We hope that this is now clearer. We have also made the colours more overlapping within model types. We have clear, expanded definitions of these types in the legend.*

5) Figure 4: I understand the full matrix showing which papers included which resistance phenotypes is provided as a supplement and is probably too large to include in the main text as it is currently shown. But featuring these small subset of outliers with a large number of DR phenotypes in the main text (Figure 4 )doesn’t seem that helpful if the goal is to give the reader a fair sense for the scope of the literature. Wouldn’t it be possible to make a heatmap figure (or similar) that shows the frequency at which different DR phenotypes are included in all models in stage 1 (not just those with >3 pheotypes) even if you can’t show all of the details about the studies in the figure?

*We take your feedback on board, noting that it was a challenge to represent this message visually. Instead of including a small section of the resistances in the main text, we have opted for a treemap focusing on all the specific resistances modelled. This should provide the reader with a visual overview of what resistances were modelled, with a more granular and detailed visualisation of all models in stage 1 in figure S1 in the supplementary.*

6) Line 406-7: “Where heterogeneity was captured, it was mostly through fitness variation”. I am unsure as what is really meant here as fitness is a composite characteristic. This is too vague a term and may differ for within host (eg differences in growth rates in absence of antibiotic pressure?) and transmission models (eg differences in transmission capacity?, differences in ability to cause disease after infection?).

*We have altered the sentence to read now “Where heterogeneity was captured in both bacterial population and between-host models, it was mostly through a variation in the model-specific fitness parameter (with the definition of fitness varying broadly from being related to transmission, ability to cause disease or speed of bacterial growth.” (lines 522-525)*

7) I have questions about the completeness of the literature search (or maybe don’t quite understand the inclusion criteria), for example work from Abel zur Wiesch seems like it should have been included?
a. [https://pubmed.ncbi.nlm.nih.gov/25972005/](https://protect-eu.mimecast.com/s/cGLYCD8vnCQ5M7YHATmuj?domain=pubmed.ncbi.nlm.nih.gov)

*This paper does not fall into our review as it is a model of E. coli. The paper also includes mycobacterium data but not a mathematical model. We have made it clearer that models that use data but do not produce DR-mycobacteria results were excluded (line 204, table 1)*

8) I think there is a missed opportunity for the authors to use the Discussion to be more explicit about the consequences of not considering bacterial heterogeneity in these types of models – the decision (implicit or explicit) to omit this type of heterogeneity means that the models don’t allow for drug treatment to operate as a selective pressure. Whether this is a problem for models depends on the specific questions being asked, the specific selection pressure being exerted, and the time frame over which the effects are considered. The title of the paper suggests that this is almost always a problem, but I think worth considering being a bit less prescriptive. As the authors rightly argue elsewhere, all these models need to make simplifying decisions, so I would tend to be careful to suggest that models of DR TB always need to include such heterogeneity.

*Thank you for making this clear statement about what we were missing. In the Introduction, we have added the following lines (114-122): “We may lose the subtlety in model outputs when modelling antibiotic treatment as a selective pressure if the traits allowing for bacterial heterogeneity are not included. Models may miss key dynamics, such as competition between strains and antibiotic effectiveness against different strains, and be at risk of predicting a treatment intervention to be more effective than it truly is. In the end, what will be included in a model will depend on the questions being asked, the selective pressures modelled, and the time frame studied.”*

*And the following lines to the discussion (580-583): “Or how including multiple levels of resistance to one antibiotic can affect treatment outcomes (44,61). Authors cannot capture all the subtlety of antibiotics as a selection pressure without including the related resistance dynamics and from this the population diversity it fosters.”*

Reviewer #2: 1. What seems to be missing in the paper is a strong justification of why it matters to account for bacterial heterogeneity in models of tuberculosis. The authors mention that predicted outcomes may differ between models that do or do not account for heterogeneity but this is somewhat of a truism (we would expect that models with different structures and/or parameters would have different results). It would be helpful to provide more specifics in terms of the hypothesized differences (e.g., would we expect such models that do not include bacterial heterogeneity to underestimate or overestimate the development of resistance or effectiveness of treatments? Does the expected magnitude of these differences justify increasing model complexity?). If there is little evidence to date as to the value of incorporating bacterial heterogeneity in these models, then that can be stated as well, and can help to justify the need for this review.

Thank you for highlighting this and suggesting changes we could make to strengthen our argument. We have made the changes below.

*(lines 114-122) “We may lose the subtlety in model outputs when modelling antibiotic treatment as a selective pressure if the traits allowing for bacterial heterogeneity are not included. Models may miss key dynamics, such as competition between strains and antibiotic effectiveness against different strains, and be at risk of predicting a treatment intervention to be more effective than it truly is. In the end, what will be included in a model will depend on the questions being asked, the selective pressures modelled, and the time-frame studied.”*

*Thank you for highlighting this. We have added a sentence in the introduction which reads: “To date, there is little evidence on how bacterial heterogeneity is incorporated into DR-TB models and little evidence of the effect this would have on model outcomes. ” on lines 133-135*

*And the following lines to the discussion (580-583): “Or how including multiple levels of resistance to one antibiotic can affect treatment outcomes (44,61). Authors cannot effectively use antibiotics as a selection pressure without including the related resistance dynamics and from this the population diversity it fosters.”*

*Lines 627-628) “…such as competition or treatment efficacy differences between strains and may, for example, under or overestimate the…”*

2. Methods, line 132-135: can you please explain why bacterial heterogeneity was defined differently for bacterial vs. human population models?

*We have expanded on this point in both the introduction and methods with the following:*

*Lines 146-160: “ We envisaged two broad settings of papers to be included in this review, within-host and between-host transmission models. This was noted by Cohen et al. (2009), a previous review of the DR-TB modelling literature (4), where “between-host” models refer to models on the human population scale. Since 2009, there has been an increase in models of bacterial populations set in the laboratory. As the populations captured will be similar to within-host models, we combined laboratory models and within-host models and collectively called them “bacterial population” models.*

*The aims, dynamics and model structure of between-host models differ considerably from bacterial population models, namely by transmission of the pathogen and populations included, making them difficult to compare. Therefore, we defined heterogeneity differently for bacterial populations and between-host models to compare methods within these categories and gain a clearer picture of bacterial heterogeneity modelling.”*

*Lines 215-220: “We split models into two groupings: “between-host” and “bacterial population” models, with the differences in their model scale, structure, and aims, resulting in a different bacterial heterogeneity definition.”*

**Part III – Minor Issues: Editorial and Data Presentation Modifications**
Please use this section for editorial suggestions as well as relatively minor modifications of existing data that would enhance clarity.

Reviewer #1: Minor comments:
1) Line 92 – the MDR/RR designation also reflects that the scale up of Xpert, which reports Rif-R resistance but not INH-R, has meant that we often know Rif-R status but not INH-R.

*We have added in “...through genotypic testing” but not added mention of INH-R as it is not the appropriate location in the introduction (line 97)*

2) Line 198 – for the second stage of literature review, how many reviewers also help to identify which of the papers met heterogeneity inclusion criteria?

*In line 274, we state that three authors determined if the models met the bacterial heterogeneity definition.*

3) Line 460 – “mathematically, it is difficult to include complexities in all aspects…” I don’t think it is the math that makes it challenging to include complexity.

*We have changed this to “Mathematically****, it can be*** *difficult…” (line 583)*

Reviewer #2: 1. It would be helpful to state clearly which aspects of bacterial heterogeneity the authors analyze in this work (e.g., heterogeneity in fitness cost of specific mutations, persistence in anatomic compartments, response to specific drugs). Stating this clearly in the Introduction will help keep the reader focused on what the primary goals of the paper are.

*Thank you for highlighting this detail, which will add clarity. We have updated our bacterial heterogeneity definitions to include more specific examples whilst noting these definitions were extracted at full-text extractions. As we did not expect many papers, we could be relatively flexible in our choice of aspects and did not focus on a single characteristic.*

2. Please consider moving some of the Results section to the appendix so that the Results focus on the main purpose of the paper, to evaluated the inclusion of bacterial heterogeneity. There is lots of other very valuable information (e.g., study settings, data sources, etc…) but presenting the results in a kind of laundry list raises the risk of readers getting lost. At the very least, I would recommend placing the results on bacterial heterogeneity first, followed by all of the other results.

*We have addressed this by removing some of the more list-like parts of the results e.g. lines 294-297 and lines 299-301)*

3. In the Discussion the authors bring up the fact that most papers incorporate resistance as a discrete value, but use continuous distributions for fitness costs. One potential reason for that is that there are point estimates for response to treatment conditional on drug resistance available in the literature or from primary data, whereas such data are rare if at all available for fitness costs (thus forcing modelers to consider a broad range of potential values across a continuous distribution).

*Thank you for raising this point. We have added in a sentence in line 605 “This contrasts with the lack of data linking resistant strain variation with treatment outcomes such as failure or recovery.”*

4. The wording used in the paper can sometimes be vague or confusing. For example, rather than referring to “different resistances”, I would suggest “types” or “patterns” of resistance. Similarly, in the Supplement, I would suggest rephrasing “actual” vs. “theoretical” resistance to “specific” vs. “hypothetical”.

*In line 137, we have changed the sentence to read “...different patterns of resistance …”. However, we are not clear on where “different resistances” is being used in the text and it is unclear.*

*We have changed the phrasing of “actual” vs. “theoretical” to “specific” vs. “hypothetical” in the main text and the supporting information as we agree this change is clearer to understand.*

5. Please consider rephrasing “data analysis” to “parameter estimation” when describing the aims of models.

*We have made this change in the main text, figure 3, and supporting information.*

6. Results, line 227: the authors state that the distribution of countries covered in the selected modeling papers does not reflect the settings with highest DR-TB incidence but this is not really true: The countries with the most modeling papers are all in the top 5 of DR-TB incidence.

*The sentence now reads “This reflects the settings with the highest MDR-TB incidence but also highlights some countries that are not being focused on” (line 308)*

7. In the legend to Figure 4, please specify that the papers listed are those considering resistance to 3 or more specific drugs (rather than just “multiple”). Also, please make sure to include the paper indices matching the Table S1 in the figure (as mentioned in the legend).

*We have updated figure 4 to reflect other comments above.*

8. Please review the supplemental materials carefully for typographical and grammatical errors.

*Thank you for pointing this out, it has been reviewed.*
