# Supplementary material for: Mathematical models of drug-resistant tuberculosis lack bacterial heterogeneity: A systematic review
Source: PLoS Pathog. 2024 Apr 10;20(4):e1011574. doi: 10.1371/journal.ppat.1011574 (PMC11060536; doi:10.1371/journal.ppat.1011574)
Supplement: Attachment — Submitted filename: Responses to Reviewer Questions 070324.docx [file ppat.1011574.s010.docx]

Reviewer Comments (if any, and for reference):

*Thank you for the additional comments – we have addressed them below, responding to each comment in italics.*

Reviewer's Responses to Questions

**Part I – Summary**
Please use this section to discuss strengths/weaknesses of study, novelty/significance, general execution and scholarship.

Reviewer #1: (No Response)

Reviewer #2: The authors have appropriately addressed most of the comments from the first round of reviews. Again, this is an impressive and valuable review of approaches used to model durg resistance in mathematical models of tuberculosis. I only have minor editorial comments to improve the clarity for readers.

*Thank you, these comments have been addressed in the text.*

**Part II – Major Issues: Key Experiments Required for Acceptance**
Please use this section to detail the key new experiments or modifications of existing experiments that should be absolutely required to validate study conclusions.

Generally, there should be no more than 3 such required experiments or major modifications for a "Major Revision" recommendation. If more than 3 experiments are necessary to validate the study conclusions, then you are encouraged to recommend "Reject".

Reviewer #1: (No Response)

Reviewer #2: (No Response)

**Part III – Minor Issues: Editorial and Data Presentation Modifications**
Please use this section for editorial suggestions as well as relatively minor modifications of existing data that would enhance clarity.

Reviewer #1: It does not seem consistent to list MDR/RR under the "multiple" drug resistant category as depicted in Figure 4, but then list models that only modeled MDR/RR as single resistance in Figure 5.

Perhaps I have misunderstood? But if not, this classification should be made consistent throughout the manuscript.

*Thank you for highlighting this inconsistency, we can see why this would be confusing to readers. The issue is that many models capture susceptible and then also “MDR/RR” resistance i.e. only a single resistance class that is comprised of multiple resistances. We have made the following changes to Figure 5 to address this:*

- *“Single resistance” to “Single resistance class”*
- *“Resistance to single antibiotic in model. […]” to “Single class of antibiotic resistance in model.”*
- *“Multi resistance” to “Multiple resistance classes”*
- *”All models have resistance to more than one antibiotic” to “All models have more than one class of antibiotic resistance”*

Reviewer #2: 1. I think that the manuscript would benefit from more clarity in the language used, especially when referring to drug resistance. Often the authors use terms such as "mutiple resistances". I would suggest selecting an alternative such as "classes of resistance", "categories of resistance" or "resistance phenotypes", and using it consistently throughout.

*Thank you for highlighting how this may be confusing to readers, we have altered the results section (lines 365, 368, 369, 374, 397, 398) and the abstract (line 56) to state “multiple resistance classes” or “single resistance class”. We have also added in the following definition of resistance class in the method, lines 222-225.*

*" A resistance class is defined as a model stratification whereby strains (or the populations including them) are grouped across multiple antibiotic resistances (i.e. MDR could here be a single “resistance class” but represents resistance to multiple antibiotic agents).“*

*Along with the comment above from reviewer 1 we have also altered figures 4 and 5 to reflect this change.*

2. Similarly, the authors could consider distinghuishing the two broad classes of models that they considered as "bacterial population" vs. "human population".

*Thank you for raising this point as we had these terms in the original manuscript. Whilst there are merits for using “human population” over “between host”, following the comments raised by reviewer 1 in the first round of reviews, we believe that this was unclear and led to some ambiguity. We suggest that the current definitions used as “bacterial population” vs “between-host” accompanied by the definitions provided in lines 193-204 give the reader clearer defining terms for the two broad model categories. In particular, we are interested in clarifying whether models include transmission of bacteria (in the “between host” models) or not (“bacterial population”). We also chose not to use the “human population” definition in part as we think this might make it unclear how to group the similar model structures that you would use for modelling a bacterial population growing in a granuloma (in a human) with that for those capturing dynamics in a test tube.*
